# 3-Bromo-2-hy­droxy­benzaldehyde

**DOI:** 10.1107/S1600536812031510

**Published:** 2012-07-18

**Authors:** Jessica B. Metlay, Joseph M. Tanski

**Affiliations:** a Department of Chemistry, Vassar College, Poughkeepsie, NY 12604, USA

## Abstract

The mol­ecule of the title compound, C_7_H_5_BrO_2_, is almost planar (r.m.s. deviation from the plane of all the non-H atoms = 0.0271 Å) and displays intra­molecular O—H⋯O hydrogen bonding between the phenol group and the aldehyde O atom. Packing is directed by weak inter­molecular C—H⋯Br inter­actions and π-stacking between nearly parallel mol­ecules [dihedral angle = 5.30 (6)° and centroid–centroid distance = 3.752 (1) Å].

## Related literature

For information on the synthesis of the title compound, see: Hansen & Skattebol (2005 ▶). For recent uses of the title compound in the synthesis of biologically active compounds, see: Velázquez *et al.* (2012 ▶); Wang *et al.* (2012 ▶); Zhang *et al.* (2012 ▶). For use of the title compound to prepare Schiff base ligands for metal coordination chemistry, see: Escudero-Adán *et al.* (2010 ▶); McGarrigle *et al.* (2004 ▶); Tzubery & Tshuva (2012 ▶). For related crystal structures, see: Balasubramani *et al.* (2011 ▶); Fan, You, Liu, Qian & Huang (2008 ▶); Fan, You, Qian, Liu & Huang (2008 ▶) Iwasaki *et al.* (1976 ▶); Kirchner *et al.* (2011 ▶); Tang *et al.* (2010 ▶).
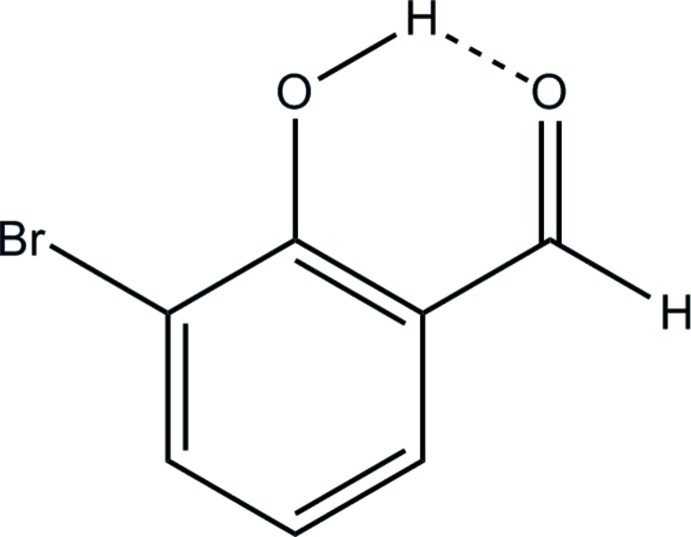


## Experimental

### 

#### Crystal data

C_7_H_5_BrO_2_
*M* *_r_* = 201.02
Monoclinic, 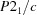

*a* = 7.0282 (3) Å
*b* = 14.9715 (7) Å
*c* = 6.8472 (3) Å
β = 108.907 (1)°
*V* = 681.61 (5) Å^3^
*Z* = 4
Mo *K*α radiation
μ = 5.96 mm^−1^
*T* = 125 K
0.22 × 0.08 × 0.03 mm

#### Data collection

Bruker APEXII CCD diffractometer
Absorption correction: multi-scan (*SADABS*; Bruker 2007 ▶) *T*_min_ = 0.354, *T*_max_ = 0.842
10788 measured reflections
2074 independent reflections
1815 reflections with *I* > 2σ(*I*)
*R*_int_ = 0.024

#### Refinement

*R*[*F* ^2^ > 2σ(*F* ^2^)] = 0.019
*wR*(*F* ^2^) = 0.048
*S* = 1.04
2074 reflections
95 parameters
H atoms treated by a mixture of independent and constrained refinement
Δρ_max_ = 0.46 e Å^−3^
Δρ_min_ = −0.25 e Å^−3^

### 

Data collection: *APEX2* (Bruker, 2007 ▶); cell refinement: *SAINT* (Bruker, 2007 ▶); data reduction: *SAINT*; program(s) used to solve structure: *SHELXS97* (Sheldrick, 2008 ▶); program(s) used to refine structure: *SHELXL97* (Sheldrick, 2008 ▶); molecular graphics: *SHELXTL* (Sheldrick, 2008 ▶); software used to prepare material for publication: *SHELXTL* and *OLEX2* (Dolomanov *et al.*, 2009 ▶).

## Supplementary Material

Crystal structure: contains datablock(s) I, global. DOI: 10.1107/S1600536812031510/bg2472sup1.cif

Structure factors: contains datablock(s) I. DOI: 10.1107/S1600536812031510/bg2472Isup2.hkl

Supplementary material file. DOI: 10.1107/S1600536812031510/bg2472Isup3.cml

Additional supplementary materials:  crystallographic information; 3D view; checkCIF report

## Figures and Tables

**Table 1:** Hydrogen-bond and C—H⋯Br interaction geometry (Å, °)

*D*—H⋯*A*	*D*—H	H⋯*A*	*D*⋯*A*	*D*—H⋯*A*
O2—H2⋯O1	0.79 (2)	1.90 (2)	2.6364 (16)	154 (2)
C4—H4⋯Br1^i^	0.95	3.05	3.798 (2)	137

Symmetry code: (i) 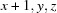.

**Fig. 1.**
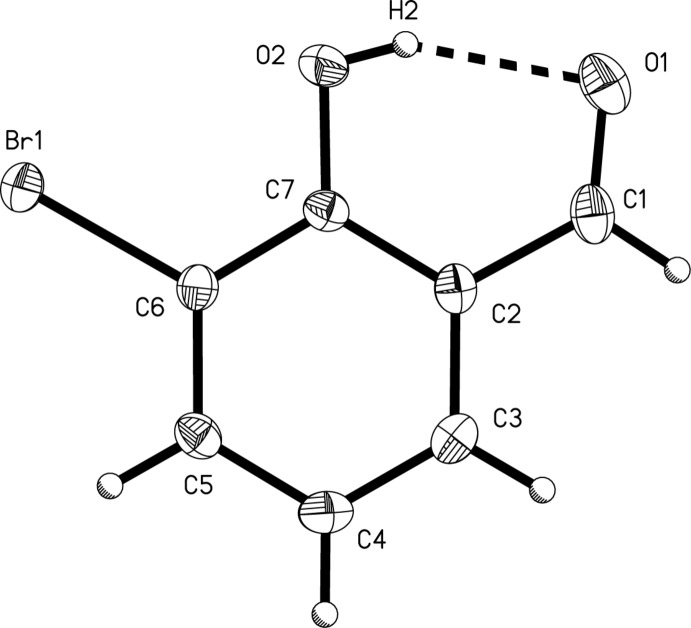
A view of the title compound with the atom numbering scheme. Displacement ellipsoids are shown at the 50% probability level.

**Fig. 2.**
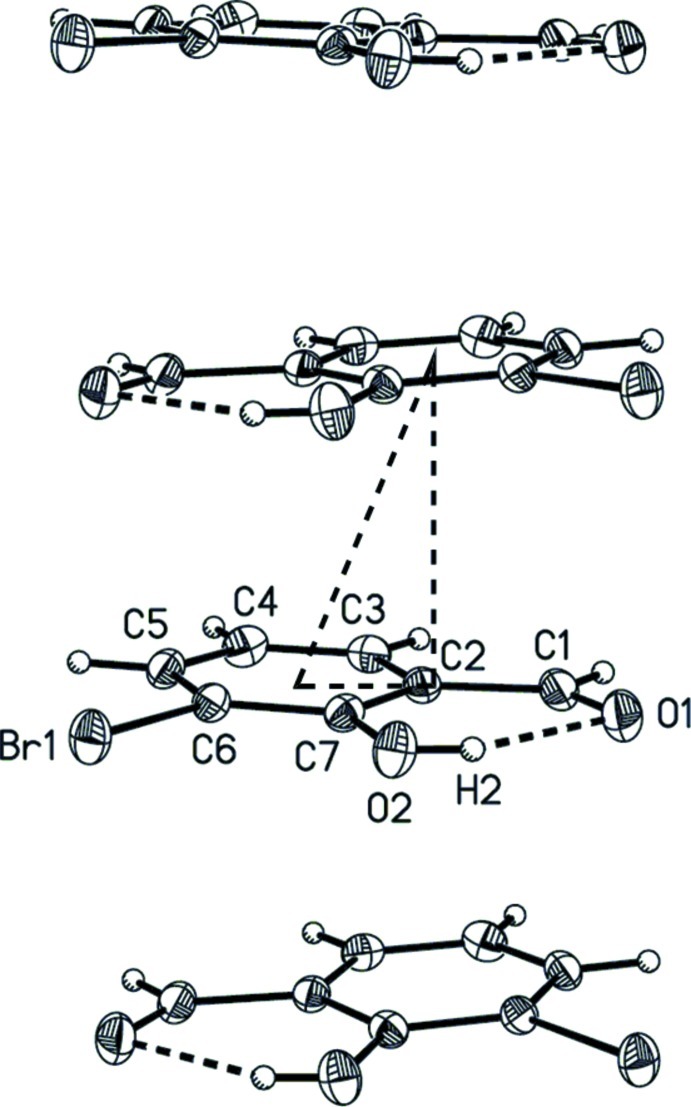
A view of the offset face-to-face π-stacking in the structure of the title compound (See text for details)

## supplementary crystallographic information

### Comment

The title compound, 3-bromo-2-hydroxybenzaldehyde, may be synthesized by reflux of 2-bromophenol with anhydrous magnesium dichloride, solid paraformaldehyde, and triethylamine in dry tetrahydrofuran (Hansen & Skattebol, 2005). Salicylaldehyde and its derivatives are commonly employed in the formation of Schiff bases for use as ligands in metal coordination chemistry. Schiff base complexes derived from 3-bromo-2-hydroxybenzaldehyde have been reported for several metals, including titanium (Tzubery & Tshuva, 2012), zinc (Escudero-Adán *et al.*, 2010) and chromium (McGarrigle *et al.*, 2004). 3-Bromo-2-hydroxybenzaldehyde is used as a synthetic reagent in the synthesis of biologically active compounds such as potential antiviral compounds (Velázquez *et al.*, 2012), chiral aromatic spiroketals (Wang *et al.*, 2012), and anticancer agents (Zhang *et al.*, 2012).

The structure of the title compound (Fig. 1) shows that the molecule is planar, with a root mean square deviation from the plane of all atoms, excluding the aryl H atoms, of 0.0271 Å. The phenol is intramolecularly hydrogen bonded to the aldehyde group *meta* to it on the aryl ring, with an O···O distance of 2.6364 (16) Å and O—H···O angle of 154 (2)°. This intramolecular hydrogen bond is common to salicylaldehyde derivatives, having metrical parameters comparable to related structures (viz., 3,5-dibromo-2-hydroxybenzaldehyde (Fan, You, Qian, Liu & Huang, 2008); 3,5-dichloro-2-hydroxybenzaldehyde (Fan, You, Liu, Qian & Huang, 2008); 3-bromo-5-*tert*-butyl-2 -hydroxybenzaldehyde (Balasubramani *et al.*, 2011); 2-hydroxy-3-methoxybenzaldehyde (Iwasaki *et al.*, 1976); 2-hydroxy-3-nitrobenzaldehyde (Tang *et al.*, 2010); hydroxybenzaldehyde (Kirchner *et al.*, 2011). While O···O distances are rather similar in these structures (range: 2.597 (3)-2.713 (6)Å; Δ: 5%), O—H···O angles are slightly more uneven (range: 143 (2)-163 (2)Å, Δ: 13%) depending on the nature of intermolecular interactions involving the phenol and aldehyde substituents on neighbouring molecules.

Inspection of the molecular packing reveals that the crystal structure is organized by weak intermolecular C-H···Br interactions, with an H···Br distance of 3.05 Å and C—H···Br angle of 136.74°. There also exists an offset face-to-face π-stacking chain of molecues running parallel to the crystallographic c-axis, with an angle between the planes of the overlapping molecules of 5.30 (6)°. This π-stacking is characterized by a centroid-to-centroid distance of 3.752 (1) Å and centroid-to-plance distances of 3.346 (1) and 3.488 (1), resulting in a ring-offsets of 1.381 (2) and 1.697 (2) Å, respectively (Fig 2).

### Experimental

Crystalline 3-bromo-2-hydroxybenzaldehyde was purchased from Aldrich Chemical Company, USA.

### Refinement

All non-hydrogen atoms were refined anisotropically. Hydrogen atoms on carbon were included in calculated positions and refined using a riding model at C–H = 0.95 Å and *U*_iso_(H) = 1.2 × *U*_eq_(C) of the aryl C-atoms. The hydrogen atom on oxygen was located in the difference map and refined freely. The extinction parameter (EXTI) refined to zero and was removed from the refinement.

### Crystal data

**Table:**

C_7_H_5_BrO_2_	*F*(000) = 392
*M**_r_* = 201.02	*D*_x_ = 1.959 Mg m^−^^3^
Monoclinic, *P*2_1_/*c*	Mo *K*α radiation, λ = 0.71073 Å
Hall symbol: -P 2ybc	Cell parameters from 6585 reflections
*a* = 7.0282 (3) Å	θ = 2.7–30.5°
*b* = 14.9715 (7) Å	µ = 5.96 mm^−^^1^
*c* = 6.8472 (3) Å	*T* = 125 K
β = 108.907 (1)°	Plate, colourless
*V* = 681.61 (5) Å^3^	0.22 × 0.08 × 0.03 mm
*Z* = 4	

### Data collection

**Table:**

Bruker APEXII CCD diffractometer	2074 independent reflections
Radiation source: fine-focus sealed tube	1815 reflections with *I* > 2σ(*I*)
Graphite monochromator	*R*_int_ = 0.024
φ and ω scans	θ_max_ = 30.5°, θ_min_ = 2.7°
Absorption correction: multi-scan (*SADABS*; Bruker 2007)	*h* = −10→10
*T*_min_ = 0.354, *T*_max_ = 0.842	*k* = −21→21
10788 measured reflections	*l* = −9→9

### Refinement

**Table:**

Refinement on *F*^2^	Primary atom site location: structure-invariant direct methods
Least-squares matrix: full	Secondary atom site location: difference Fourier map
*R*[*F*^2^ > 2σ(*F*^2^)] = 0.019	Hydrogen site location: inferred from neighbouring sites
*wR*(*F*^2^) = 0.048	H atoms treated by a mixture of independent and constrained refinement
*S* = 1.04	*w* = 1/[σ^2^(*F*_o_^2^) + (0.0249*P*)^2^ + 0.1779*P*] where *P* = (*F*_o_^2^ + 2*F*_c_^2^)/3
2074 reflections	(Δ/σ)_max_ = 0.001
95 parameters	Δρ_max_ = 0.46 e Å^−^^3^
0 restraints	Δρ_min_ = −0.25 e Å^−^^3^

### Special details

**Table:**

Geometry. All e.s.d.'s (except the e.s.d. in the dihedral angle between two l.s. planes) are estimated using the full covariance matrix. The cell e.s.d.'s are taken into account individually in the estimation of e.s.d.'s in distances, angles and torsion angles; correlations between e.s.d.'s in cell parameters are only used when they are defined by crystal symmetry. An approximate (isotropic) treatment of cell e.s.d.'s is used for estimating e.s.d.'s involving l.s. planes.
Refinement. Refinement of *F*^2^ against ALL reflections. The weighted *R*-factor *wR* and goodness of fit *S* are based on *F*^2^, conventional *R*-factors *R* are based on *F*, with *F* set to zero for negative *F*^2^. The threshold expression of *F*^2^ > σ(*F*^2^) is used only for calculating *R*-factors(gt) *etc*. and is not relevant to the choice of reflections for refinement. *R*-factors based on *F*^2^ are statistically about twice as large as those based on *F*, and *R*- factors based on ALL data will be even larger.

### Fractional atomic coordinates and isotropic or equivalent isotropic displacement parameters (Å^2^)

**Table:**

	*x*	*y*	*z*	*U*_iso_*/*U*_eq_	
Br1	0.11374 (2)	0.567091 (9)	0.25208 (2)	0.02342 (6)	
O2	0.12613 (15)	0.76969 (8)	0.28306 (17)	0.0236 (2)	
H2	0.144 (4)	0.8217 (17)	0.294 (4)	0.055 (8)*	
C1	0.4646 (2)	0.88799 (9)	0.3840 (2)	0.0228 (3)	
H1	0.5839	0.9229	0.4229	0.027*	
O1	0.30259 (19)	0.92736 (7)	0.33528 (18)	0.0284 (2)	
C6	0.3410 (2)	0.64329 (9)	0.3299 (2)	0.0170 (2)	
C7	0.3132 (2)	0.73579 (9)	0.3323 (2)	0.0166 (2)	
C3	0.6776 (2)	0.75351 (10)	0.4401 (2)	0.0202 (3)	
H3	0.7925	0.7913	0.4781	0.024*	
C5	0.5322 (2)	0.60728 (9)	0.3814 (2)	0.0192 (3)	
H5	0.5480	0.5443	0.3776	0.023*	
C2	0.4853 (2)	0.79087 (9)	0.38599 (19)	0.0177 (2)	
C4	0.7016 (2)	0.66178 (10)	0.4385 (2)	0.0219 (3)	
H4	0.8323	0.6363	0.4760	0.026*	

### Atomic displacement parameters (Å^2^)

**Table:**

	*U*^11^	*U*^22^	*U*^33^	*U*^12^	*U*^13^	*U*^23^
Br1	0.01950 (8)	0.01875 (8)	0.03050 (9)	−0.00399 (5)	0.00602 (6)	−0.00323 (5)
O2	0.0177 (5)	0.0199 (5)	0.0302 (6)	0.0043 (4)	0.0037 (4)	−0.0004 (4)
C1	0.0317 (8)	0.0177 (6)	0.0188 (6)	−0.0035 (6)	0.0080 (6)	0.0005 (5)
O1	0.0383 (7)	0.0187 (5)	0.0275 (6)	0.0039 (4)	0.0095 (5)	0.0031 (4)
C6	0.0183 (6)	0.0165 (6)	0.0158 (6)	−0.0017 (5)	0.0049 (5)	−0.0020 (5)
C7	0.0168 (6)	0.0177 (6)	0.0144 (6)	0.0022 (5)	0.0040 (5)	−0.0002 (5)
C3	0.0190 (7)	0.0241 (7)	0.0175 (6)	−0.0041 (5)	0.0059 (5)	−0.0014 (5)
C5	0.0222 (7)	0.0172 (6)	0.0181 (6)	0.0027 (5)	0.0062 (5)	−0.0014 (5)
C2	0.0222 (6)	0.0169 (6)	0.0134 (6)	−0.0018 (5)	0.0050 (5)	−0.0005 (5)
C4	0.0174 (6)	0.0265 (7)	0.0208 (6)	0.0037 (5)	0.0048 (5)	−0.0009 (5)

### Geometric parameters (Å, º)

**Table:**

Br1—C6	1.8933 (13)	C7—C2	1.4107 (19)
O2—C7	1.3466 (16)	C3—C4	1.384 (2)
O2—H2	0.79 (2)	C3—C2	1.397 (2)
C1—O1	1.2284 (19)	C3—H3	0.9500
C1—C2	1.4609 (19)	C5—C4	1.391 (2)
C1—H1	0.9500	C5—H5	0.9500
C6—C5	1.3836 (19)	C4—H4	0.9500
C6—C7	1.3993 (19)		
			
C7—O2—H2	103.8 (18)	C4—C3—H3	119.8
O1—C1—C2	124.12 (14)	C2—C3—H3	119.8
O1—C1—H1	117.9	C6—C5—C4	121.02 (13)
C2—C1—H1	117.9	C6—C5—H5	119.5
C5—C6—C7	120.67 (13)	C4—C5—H5	119.5
C5—C6—Br1	119.87 (10)	C3—C2—C7	120.60 (13)
C7—C6—Br1	119.45 (10)	C3—C2—C1	119.08 (13)
O2—C7—C6	119.90 (12)	C7—C2—C1	120.32 (13)
O2—C7—C2	122.02 (12)	C3—C4—C5	119.28 (13)
C6—C7—C2	118.08 (12)	C3—C4—H4	120.4
C4—C3—C2	120.32 (13)	C5—C4—H4	120.4
			
C5—C6—C7—O2	−179.59 (12)	O2—C7—C2—C3	178.81 (12)
Br1—C6—C7—O2	1.18 (17)	C6—C7—C2—C3	−1.59 (19)
C5—C6—C7—C2	0.79 (19)	O2—C7—C2—C1	−1.71 (19)
Br1—C6—C7—C2	−178.43 (9)	C6—C7—C2—C1	177.89 (12)
C7—C6—C5—C4	0.6 (2)	O1—C1—C2—C3	178.64 (13)
Br1—C6—C5—C4	179.81 (11)	O1—C1—C2—C7	−0.9 (2)
C4—C3—C2—C7	1.0 (2)	C2—C3—C4—C5	0.4 (2)
C4—C3—C2—C1	−178.47 (13)	C6—C5—C4—C3	−1.2 (2)

### Hydrogen-bond geometry (Å, º)

**Table:**

*D*—H···*A*	*D*—H	H···*A*	*D*···*A*	*D*—H···*A*
O2—H2···O1	0.79 (2)	1.90 (2)	2.6364 (16)	154 (2)
C4—H4···Br1^i^	0.95	3.05	3.798 (2)	137

Symmetry code: (i) *x*+1, *y*, *z*.
